# Functional near-infrared spectroscopy pre-ictal oscillations recorded simultaneously with stereo-EEG

**DOI:** 10.1016/j.ynirp.2026.100401

**Published:** 2026-09-10

**Authors:** Netaniel Rein, Revital Shechter, Yael Avni, Evgeny Tsizin, Oshrit Arviv, Dana Ekstein, Tal Benoliel, Diya Doufish, Tal Gilboa, Naomi Froimovich, Yuliya Katz, Paz Har-Shai Yahav, Guy Rosenthal, Sami Heymann, Zvi Israel, Michal Balberg, Mordekhay Medvedovsky

**Affiliations:** a Department of Neurology and Agnes Ginges Center for Human Neurogenetics, Hadassah Medical Organization, Jerusalem, Israel; b The Faculty of Medicine, Hebrew University of Jerusalem, Jerusalem, Israel; c Neuro-Electro-Light Center (NELC), Hadassah Medical Organization and Holon Institute of Technology, Israel; d Faculty of Engineering, Holon Institute of Technology, Holon, Israel; e Pediatric Neurology Unit, Hadassah University Hospital, Jerusalem, Israel; f Department of Neurosurgery, Hadassah Medical Organization, Jerusalem, Israel

**Keywords:** fNIRS-SEEG, Pre-ictal detection, Functional neuroimaging, CWT, Very low frequency hemodynamic oscillations

## Abstract

**Background:**

Peri-ictal hemodynamic alterations have been consistently demonstrated using functional near-infrared spectroscopy (fNIRS). However, whether ultra-slow hemodynamic activity develops before intracranially defined seizure onset remains uncertain. We investigated this question using simultaneous stereotactic EEG (SEEG) and fNIRS recordings, providing a precise intracranial temporal reference for seizure onset.

**Objective:**

To investigate whether ultra-slow hemodynamic activity precedes SEEG-defined seizure onset and occurs more frequently than expected by chance in prolonged recordings.

**Methods:**

Five patients undergoing simultaneous SEEG-fNIRS monitoring contributed 16 analyzable seizures. Exploratory inspection of seizure-averaged continuous wavelet scalograms identified a candidate ultra-slow frequency band(0.002–0.004 Hz), which was subsequently evaluated using a predefined permutation framework comparing true seizures with pseudo-seizure events sampled from the same recordings. Statistical inference combined channel-level permutation testing with seizure-level permutation-based Fisher analyses, supported by spatial and short-separation control analyses.

**Results:**

Evidence for ultra-slow hemodynamic activity preceding SEEG-defined seizure onset was identified in three of five patients and in 10 of 16 analyzable seizures. One long-separation channel remained significant following family-wise error correction, while multiple seizures demonstrated significant permutation-based Fisher analyses. Nominally significant channels frequently formed spatial clusters that broadly corresponded to independently defined seizure onset hypotheses and were generally not mirrored by short-separation channels. Temporal centers of gravity, used as conservative descriptors of the temporal distribution of activity, preceded SEEG-defined seizure onset by 72.0–632.1 s for HbO and 20.3–592.0 s for HbR, consistent with a substantial pre-ictal component.

**Conclusions:**

Ultra-slow hemodynamic activity frequently developed before and preferentially around SEEG-defined seizure onset compared with pseudo-seizure events, suggesting these fluctuations are unlikely to reflect chance occurrences within prolonged recordings. Although the physiological origin and clinical significance of these oscillations remain uncertain, and the analyzed frequency band should be regarded as exploratory pending independent validation, convergent channel- and seizure-level analyses, spatial organization, and limited correspondence with superficial signals support further investigation of ultra-slow hemodynamic dynamics as a component of seizure-related physiology.

## Highlights

• Simultaneous SEEG-fNIRS reveals ultra-slow peri-ictal dynamics.
• Hemodynamic activity preferentially emerged near true seizure onsets.
• Pre-onset components were found in 10 of 16 analyzable seizures.
• Permutation and short-channel controls support seizure association.

## 1 Introduction

Epilepsy is a common chronic neurological condition affecting approximately seventy million individuals worldwide, with up to one-third developing drug-resistant epilepsy (DRE) (Auvin et al., 2023). One of the major challenges in epilepsy research is the identification of physiological changes that precede overt electrographic and clinical seizure onset. Reliable detection of such pre-ictal changes could improve seizure prediction, facilitate timely intervention, provide insights into seizure generation, and potentially contribute to localization of epileptogenic networks (Gadhoumi et al., 2016; Litt and Echauz, 2002; Meisel and Loddenkemper, 2020; Mormann et al., 2007).

Several modalities have demonstrated physiological alterations preceding or accompanying seizure onset, including EEG (Mormann et al., 2007), functional MRI (Vinette et al., 2016), SPECT (Baumgartner et al., 1998), and fNIRS (Nguyen et al., 2012, 2013; Peng et al., 2016; Seyal, 2014). Collectively, these findings suggest that seizure generation may involve physiological processes evolving over timescales of minutes rather than seconds. In fNIRS studies specifically, peri-ictal alterations in oxyhemoglobin (HbO) and deoxyhemoglobin (HbR) have been reported during simultaneous EEG-fNIRS monitoring (Kassab et al., 2021), including complex oxygenation patterns during temporal and frontal lobe seizures (Nguyen et al., 2012, 2013), regional oxygenation changes preceding seizure onset (Seyal, 2014), and discrimination of pre-ictal from interictal states using machine-learning approaches (Guevara et al., 2019). These observations raise the possibility that seizure-associated hemodynamic changes may extend beyond the immediate onset period and evolve over timescales substantially longer than those typically associated with epileptic discharges, although their temporal organization at ultra-low frequencies remains incompletely characterized.

However, interpretation of such findings remains challenging. Ultra-slow hemodynamic fluctuations are common in prolonged recordings and may arise from resting-state vascular oscillations, systemic physiological processes or spontaneous neurovascular activity unrelated to seizures (Obrig, 2014; Scholkmann et al., 2014; Tong and Frederick, 2014). Consequently, an important unresolved question is not simply whether very low-frequency hemodynamic oscillations occur around seizures, but whether they occur preferentially around true seizure onsets beyond what would be expected from the background fluctuations present throughout long-duration recordings.

A major challenge in addressing these questions is statistical rather than purely physiological. Ultra-slow fluctuations are prolonged, spatially correlated and non-stationary, and similar events may occur spontaneously throughout long recordings. Apparent peri-ictal changes may therefore emerge by chance unless compared against an appropriate null model. To address this issue, we employed a permutation framework based on pseudo-seizure events sampled from the same recordings. Such approaches have been widely recommended for the analysis of non-stationary neurophysiological data because they preserve the temporal structure of the underlying signal while providing an empirical estimate of chance-level effects (Maris and Oostenveld, 2007; Nichols and Holmes, 2002).

In the present study, we combined prolonged fNIRS monitoring with stereotactic EEG (SEEG), allowing seizure timing to be defined using intracranial electrophysiological recordings. The use of SEEG is particularly relevant in studies of pre-ictal physiology, as seizure onset may be detected earlier with intracranial recordings than with scalp EEG (Barborica et al., 2021), reducing the likelihood that early ictal activity is misclassified as pre-ictal.

We hypothesized that seizure-associated very low-frequency hemodynamic oscillations would occur preferentially around true seizure onsets and would be observed more frequently than expected from randomly sampled pseudo-seizure events. To test this hypothesis, we applied continuous wavelet transform (CWT), permutation-based significance testing, spatial clustering analyses, short-separation channel comparisons and seizure-level omnibus statistics to prolonged SEEG-fNIRS recordings.

## 2 Methods

This study was approved by the Hadassah Medical Center Helsinki Committee (approval number 0068-22-HMO; approved on 12 June 2022). The study was conducted in accordance with the Declaration of Helsinki and all applicable institutional regulations. Written informed consent was obtained from all participants prior to study participation. Participants also provided consent for the publication of anonymized clinical, demographic, and neurophysiological data.

**Fig. 1:**
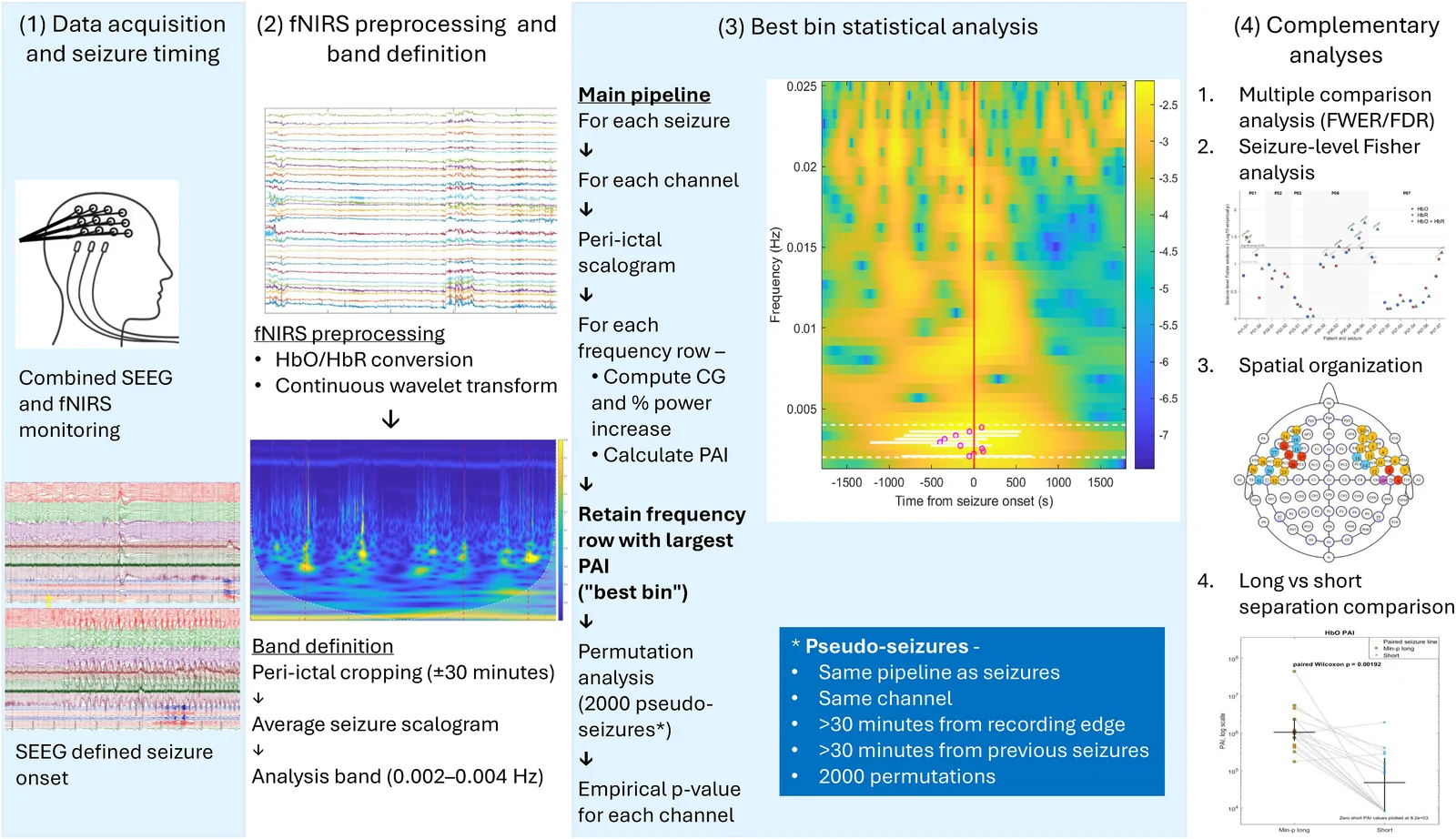
**Overview of the analysis pipeline.** Simultaneous SEEG–fNIRS recordings were acquired, and SEEG-defined seizure onset times were used as the temporal reference for all analyses. Following fNIRS preprocessing, seizure-averaged scalograms were inspected to define the 0.002–0.004 Hz analysis band. For each seizure, long-separation channel, and frequency row, the temporal center of gravity (CG), percentage power increase relative to baseline, and pre-ictal activity index (PAI) were calculated. The frequency row yielding the largest PAI ("best bin") was retained and compared with patient-specific null distributions generated from 2000 pseudo-seizure events to obtain empirical channel-level *p*-values. These results were subsequently used for multiple-comparison correction, seizure-level Fisher analyses, spatial analyses, and comparisons with short-separation channels.

A schematic overview of the analysis protocol is shown in Fig. 1.

### 2.1 Participants

Patients diagnosed with focal epilepsy who were admitted for SEEG monitoring at Hadassah Medical Center between December 2022 and January 2024 were recruited after providing informed consent. Consecutive eligible patients were recruited to minimize selection bias. Inclusion criteria were age ≥18 years and drug-resistant focal epilepsy requiring clinically indicated SEEG monitoring. The sole exclusion criterion was refusal to participate. Ethnicity and socioeconomic status were not systematically collected as part of this observational study and are therefore not reported.

fNIRS monitoring was initiated on the day following SEEG electrode implantation, after completion of the neurosurgical procedure and transfer of the patient to routine postoperative monitoring in the intensive care unit. Optode placement was performed in coordination with the neurosurgical and EEG teams and did not require modification of the implanted SEEG electrodes or surgical procedure. The setup was performed in a non-sterile clinical environment, analogous to the placement of scalp EEG electrodes during routine post-implantation monitoring, with the optodes positioned beneath the head wrap without disturbing the implanted SEEG electrodes. fNIRS recording continued until SEEG monitoring was discontinued, the participant requested termination of fNIRS recording, or clinical requirements (e.g., imaging studies or patient care procedures) prevented further recording.

### 2.2 SEEG recordings and seizure timing

SEEG data were recorded using a Natus Quantum amplifier (Natus Neurology) at 2048 Hz and 16-bit resolution. Depth electrodes were implanted according to the clinical hypothesis regarding the seizure onset zone. Seizure onset was determined from the SEEG recordings by an expert epileptologist blinded to the fNIRS data. These SEEG-defined onset times were used as the temporal anchor for all peri-ictal fNIRS analyses.

### 2.3 fNIRS recordings

fNIRS data were acquired using continuous-wave fNIRS systems. Initially, recordings were performed with the ETG-4000 Optical Topography System (Hitachi Medical Corporation, Tokyo, Japan), which emits light at 690 and 830 nm and includes ten emitters and eight detectors. Later recordings were performed using the NIRScout system (NIRx Medizintechnik GmbH, Berlin, Germany), which emits light at 785 and 830 nm and includes twelve emitters and twelve detectors. The sampling rate was 10 Hz for the ETG-4000 system and 5.2 Hz for the NIRScout system. Data was recorded for 4 h segments, or partitioned into 4 h segments for consistent comparison between the two systems (since ETG-4000 maximal file size is 4 h long).

For standard long-separation channels, emitters and detectors were positioned approximately 3 cm apart. Short-separation channels, used to assess superficial hemodynamic fluctuations, were positioned with an emitter-detector separation of approximately 1 cm. fNIRS channels were arranged in patient-specific montages guided by the clinical hypothesis regarding the seizure onset zone and constrained by the position of SEEG electrodes, dressings and clinical accessibility.

### 2.4 Signal processing

#### 2.4.1 Conversion to hemoglobin concentration changes

Using MATLAB R2024a (MathWorks, Natick, MA, USA), changes in oxyhemoglobin (HbO) and deoxyhemoglobin (HbR) concentrations were calculated using the modified Beer-Lambert law. A source-detector distance of 3 cm and a differential pathlength factor of 6 were used for long-separation channels. The initial 10-s baseline from each 4 h recording segment was used for conversion of optical intensity to relative hemoglobin concentration changes. This baseline was used only for signal conversion and should be distinguished from the separate local baseline used later for wavelet-power comparisons.

#### 2.4.2 Continuous wavelet decomposition

Following preprocessing, continuous wavelet transform (CWT) analysis was applied to all useable fNIRS channels. CWT was performed using MATLAB's analytic Morse wavelet with a time-bandwidth product of 60 and 10 voices per octave.

Continuous wavelet transform was selected because it provides a time-frequency representation well suited to non-stationary physiological signals. Its multi-resolution properties provide improved frequency resolution at ultra-low frequencies while preserving temporal localization, making it particularly suitable for characterizing peri-ictal hemodynamic dynamics evolving over several minutes.

#### 2.4.3 Seizure inclusion and peri-ictal cropping

For each SEEG-defined seizure, a peri-ictal window extending 30 min before to 30 min after seizure onset was extracted. Seizures were excluded if the required peri-ictal window extended outside the boundaries of the 4-h segment or if seizure onset occurred less than 30 min after a preceding seizure. These criteria were applied to avoid contamination from edge effects (Torrence and Compo, 1998), incomplete peri-ictal windows and overlapping peri-ictal activity from closely spaced seizures.

Analyses were performed separately for HbO and HbR. An exploratory examination of seizure-averaged peri-ictal scalograms was performed to define a candidate frequency band for formal statistical evaluation. This inspection was based solely on seizure-averaged representations and was used only to determine the frequency range carried forward into the predefined statistical pipeline. It played no role in channel selection or statistical inference. All subsequent statistical inference was performed independently across every analyzable channel within the selected frequency band using the predefined permutation framework. Accordingly, the frequency-band selection should be regarded as exploratory and hypothesis-generating.

### 2.5 Identification of pre-ictal activity

For each frequency row within the 0.002–0.004 Hz range identified in the previous step, contiguous regions of elevated wavelet power were identified using a threshold equal to the mean power of that row across the analyzed peri-ictal window. Only above-threshold regions containing seizure onset were retained, and regions shorter than 5 s were discarded.

For each onset-containing wavelet region, the temporal centre of gravity (CG) was calculated as the median power-weighted time point within the region, defined as -

$$\text{CG}=\min\left\{t\in \left[t_{\text{start}},t_{\text{end}}\right]\;|\;\sum_{i=t_{\text{start}}}^{t}P_{i}\geq \frac{1}{2}\sum_{i=t_{\text{start}}}^{t_{\text{end}}}P_{i}\right\}$$

where (P_i_) denotes the wavelet power at time point (i). Thus, the CG corresponds to the earliest time at which one-half of the cumulative wavelet power within the identified peri-ictal region has been accumulated. This definition intentionally provides a robust, conservative descriptor of the temporal distribution of activity rather than an estimate of the physiological onset of the underlying process. Conceptually, the CG represents the temporal center of mass of the oscillatory activity contained within the region.

If the CG occurred after seizure onset, the frequency row was not considered to contain pre-ictal activity and was assigned a value of zero for statistical analysis. If the CG occurred before seizure onset, the interval between CG and seizure onset was calculated:

Δt=SO−CG

where (SO) denotes SEEG-defined seizure onset and (CG) denotes the temporal center of gravity of wavelet power.

The CG therefore serves two roles: it defines the pre-ictal interval used for quantification using a conservative timing metric (see *Supplementary* Section *II*), and it provides a descriptive measure of the temporal distribution of peri-ictal fNIRS activity relative to SEEG-defined seizure onset.

#### 2.5.1 Local baseline and percentage power increase

For each seizure, channel and frequency row, baseline wavelet power was calculated from the first 500 s of the extracted peri-ictal window. This local baseline was used only for statistical quantification of wavelet-power changes and was distinct from the 10-s baseline used for Beer-Lambert conversion described above. A local baseline was selected to provide a representative reference level from the beginning of the extracted peri-ictal window while minimizing the influence of slower fluctuations occurring over longer portions of the recording. It also avoided using the mean of the entire peri-ictal window as the reference, which could attenuate prolonged ultra-slow oscillations by allowing the activity under investigation to contribute substantially to its own reference value.

For frequency rows in which the CG preceded seizure onset, the mean wavelet power between CG and seizure onset was calculated and expressed as percentage increase relative to the local baseline:

$$\Delta {\text{Power}}_{\left\{\%\right\}}=100\times \frac{\left\{{\text{Power}}_{\left\{\text{CG}\to \text{SO}\right\}}-{\text{Power}}_{\left\{\text{baseline}\right\}}\right\}}{\left\{{\text{Power}}_{\left\{\text{baseline}\right\}}\right\}}$$

#### 2.5.2 Pre-ictal activity index

The primary metric for all statistical analyses was the pre-ictal activity index (PAI), defined as:

$$\text{PAI}=\Delta t\times \Delta {\text{Power}}_{\left\{\%\right\}}$$

This metric was designed to capture both the duration and magnitude of pre-ictal activity. A brief but high-amplitude fluctuation and a prolonged but weak fluctuation may each be difficult to interpret in isolation. The PAI was therefore used to quantify activity that was both sustained before seizure onset and elevated relative to local baseline.

#### 2.5.3 Best-bin frequency analysis

The 0.002–0.004 Hz range contains multiple individual CWT frequency rows. Rather than averaging wavelet power across this range before calculating the statistic, for every seizure and channel, the PAI was calculated separately for each frequency row within 0.002–0.004 Hz. The row yielding the largest PAI value was retained as the representative statistic for that seizure-channel pair. This was termed the "best-bin" approach. This approach was used to reduce dependence on arbitrary averaging across adjacent ultra-low-frequency rows while allowing the exact frequency of maximal peri-ictal activity to vary slightly across seizures and channels.

In addition to the primary analyses, the interval between the temporal center of gravity (CG) and SEEG-defined seizure onset was summarized descriptively. For each seizure and chromophore, the CG-to-seizure-onset duration associated with the channel yielding the lowest empirical p-value was recorded. These values were not used for statistical testing but were reported to characterize the temporal scale of the observed peri-ictal activity.

### 2.6 Statistical analysis

#### 2.6.1 Permutation framework

To evaluate whether observed peri-ictal activity exceeded that expected by chance, a pseudo-seizure permutation framework was implemented. Candidate pseudo-onsets were sampled from the same recording as the real seizures. Candidate locations were excluded if they were within 30 min of a real seizure or within 30 min of a recording boundary. Remaining candidate pseudo-onsets were sampled on a 30-s grid.

For each permutation, a pseudo-onset was selected from the eligible pool and the same analysis pipeline was applied, including onset-containing region identification, peri-ictal cropping, CG calculation, PAI calculation and best-bin selection. This generated patient-specific null distributions representing the expected statistics when seizure timing is replaced by pseudo-event timing sampled from seizure-free portions of the same recording.

A total of 2000 permutations were generated for each patient and chromophore.

Empirical p-values were calculated as:

$$\frac{p=\left\{1+\#\left(T_{\left\{\text{perm}\right\}}\geq T_{\left\{\text{real}\right\}}\right)\right\}}{\left\{1+N_{\left\{\text{perm}\right\}}\right\}}$$

Where $T_{\left\{\text{real}\right\}}$ is the observed statistic, $T_{\left\{\text{perm}\right\}}$ denotes values from the permutation distribution and $N_{\left\{\text{perm}\right\}}$ is the number of permutations.

All analyzable channels were evaluated using the same predefined analytical pipeline. The identical pipeline, including best-bin selection, was applied to both true and pseudo-seizure events.

#### 2.6.2 Channel-level significance and correction for multiple comparisons

For each seizure, channel-level nominal (i.e., uncorrected) p-values were obtained by comparing the observed channel-specific PAI with the corresponding empirical null distribution from the same channel and chromophore.

To correct for multiple comparisons across channels, two complementary procedures were applied. First, false discovery rate (FDR) correction was performed using the Benjamini-Hochberg procedure. Second, family-wise error rate (FWER) correction was performed using a permutation-derived maximum-statistic approach. For each permutation, the maximal statistic across channels was retained, generating a null distribution of maximal values. Each observed channel statistic was then compared with this maximum-statistic null distribution to obtain an FWER-corrected p-value. Nominal, FDR-corrected and FWER-corrected findings are reported.

#### 2.6.3 Seizure-level Fisher analysis

Because seizure-related activity may be distributed across multiple channels without any single channel surviving strict correction, a seizure-level Fisher analysis was performed. This analysis tested whether the number and magnitude of low channel-level p-values within a seizure exceeded that expected under the permutation-derived null distribution.

For each seizure, channel-level empirical p-values were combined using Fisher's method:

$$X^{2}=-2\sum_{\left\{i=1\right\}}^{\left\{k\right\}}\;\ln\left(p_{i}\right)$$

where $p_{i}$ denotes the nominal p-value for channel (i), and (k) denotes the number of analyzed channels.

Because fNIRS channels are spatially correlated and the resulting Fisher statistic cannot be assumed to follow the standard chi-square distribution, significance was determined empirically. For each permutation, the same Fisher procedure was applied to the corresponding permutation-derived channel-level p-values, generating a seizure-specific null distribution of Fisher statistics. The observed seizure-level Fisher statistic was then compared with this empirical null distribution to obtain a seizure-level p-value.

This analysis was performed separately for HbO and HbR. A combined HbO + HbR analysis was also performed by pooling channel-level p-values across both chromophores before Fisher aggregation. The combined analysis was used to evaluate whether evidence for peri-ictal activity was strengthened when both hemodynamic signals were considered together.

### 2.7 Spatial distribution of nominal effects

To assess whether nominally significant findings were spatially organized rather than randomly distributed across the montage, channels with uncorrected p-values below 0.05 were mapped onto each patient's fNIRS layout. The spatial distribution of these channels was examined descriptively, with particular attention to whether significant channels formed contiguous clusters or appeared diffusely across the montage.

This analysis was descriptive and was not used to claim definitive localization of the seizure onset zone.

### 2.8 Short-separation channel analysis

Because only one or two short-separation channels were available per patient, owing to clinical constraints on optode placement during SEEG monitoring, these channels were not used for regression-based removal of superficial signals. Instead, they were analyzed independently as a control for superficial hemodynamic activity.

For each seizure and chromophore, the long-separation channel demonstrating the minimum empirical p-value was paired with the anatomically corresponding short-separation channel whenever available. When no corresponding short-separation channel could be assigned, the short-separation channel demonstrating the largest PAI value was used. PAI values from paired long- and short-separation channels were then compared across seizure-chromophore pairs using paired Wilcoxon signed-rank tests.

## 3 Results

### 3.1 Participant and recording characteristics

Eight consecutive adult patients with focal epilepsy undergoing clinical SEEG monitoring were recruited. Three patients were excluded from the final analysis: two because no seizures occurred during the fNIRS recording period and one because consent was withdrawn during fNIRS setup. The remaining five patients contributed analyzable data. The five analyzed participants comprised four men and one woman, aged 30–49 years, with epilepsy durations ranging from 1 to 27 years (Table 1). Three patients (P01–P03) were monitored using the Hitachi ETG-4000 system, whereas two patients (P06–P07) were monitored using the NIRScout system. Across the cohort, 28 seizures were recorded during 14–92 h of combined fNIRS–SEEG monitoring. Following application of predefined quality-control criteria, 16 seizures were retained for analysis (range: 1–6 useable seizures per patient). Recording coverage differed substantially between systems, with the Hitachi montage providing five long-separation channels and the NIRScout montage providing 30–31 long-separation channels in addition to short-separation channels. Demographic, clinical, and recording characteristics are summarized in Table 1.

**Table 1:** Demographic, clinical and recording characteristics

Patient	Sex	Age (years)	Type of epilepsy	Years with epilepsy	Seizure onset zone hypothesis	fNIRS system	Long-separation channels	Short-separation channels	Monitoring duration (h)	Seizures (recorded/analyzed)
P01	M	49	Focal impaired-awareness	1	Left frontal (cavernoma-associated)	Hitachi ETG-4000	5	1	22	6/2
P02	M	30	Focal impaired-awareness	17	Right insular/perisylvian (SEEG + stimulation)	Hitachi ETG-4000	5	1	18	5/2
P03	M	31	Focal epilepsy	26	Right parietal	Hitachi ETG-4000	5	1	14	2/1
P06	M	35	Focal impaired-awareness	27	Right medial frontal/orbitofrontal	NIRScout	30	2	92	8/5
P07	F	32	Focal epilepsy	21	Suspected right temporal	NIRScout	31	2	47.5	7/6
Total/Range	4 M, 1 F	30–49		1–27			5–31	1-2	14–92	28/16

Useable seizures were selected after eliminating seizures that started within 30 min of the borders of the 4 h recording segment or of a previous seizure.Structured.

### 3.2 Exploratory identification of ultra-slow peri-ictal activity

**Fig. 2:**
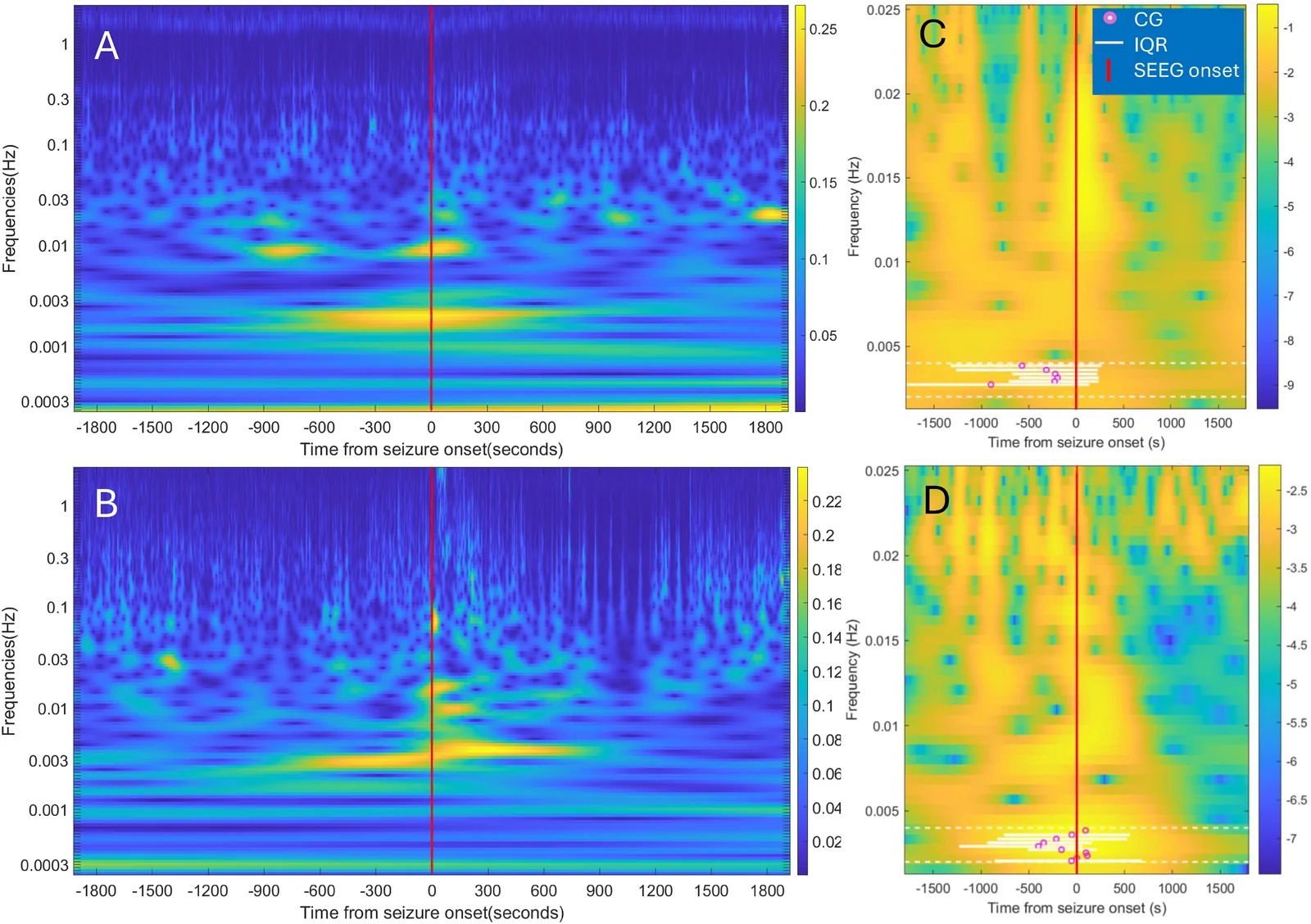
**Exploratory identification of the ultra-slow analysis band and implementation of the temporal center of gravity (CG) analysis.** (A–B) Continuous wavelet transform (CWT) scalograms demonstrating pre-ictal activity within the ultra-slow frequency range. These scalograms, averaged across seizures per patient, were used solely to identify the candidate analysis band (0.002–0.004 Hz) for subsequent evaluation and were not used for channel selection or statistical inference. Representative examples are shown for HbO (panel A, patient P06, channel 23) and HbR (panel B, patient P07, channel 31), with the horizontal red line marking SEEG defined seizure onset in all panels. (C–D) Representative examples of CG analysis for individual frequency. For each frequency row, supra-threshold wavelet activity containing SEEG-defined seizure onset was identified, and the temporal center of gravity (CG; pink circle) was calculated as the power-weighted temporal center of the detected activity. The white horizontal line indicates the interquartile range (IQR) of the above-threshold wavelet activity. The interval between CG and seizure onset was subsequently used for calculation of the pre-ictal activity index (PAI). As described in the *Methods*, CG is presented as a conservative descriptor of the temporal distribution of peri-ictal activity rather than an estimate of the physiological onset of the underlying process.

Exploratory inspection of seizure-averaged peri-ictal CWT scalograms suggested that recurrent peri-ictal activity was most consistently represented within approximately 0.002–0.004 Hz across patients (Fig. 2A–B). This observation was used to define the frequency band for subsequent formal statistical analysis but was not itself subjected to statistical inference. Although individual seizures exhibited activity over a broader range of ultra-low frequencies, the 0.002–0.004 Hz band showed the most consistent seizure-averaged peri-ictal patterns across patients and across both HbO and HbR.

### 3.3 Channel-level permutation analysis

Application of the best-bin permutation framework identified nominally significant channel-level findings in three of the five analyzed patients (P01, P06 and P07) (Table 2). In contrast, little evidence of seizure-associated activity was observed in P02 and P03.

**Table 2:** Summary of channel-level permutation findings by patient and seizure.

HbR PAI of min p channel^b^	HbR CG→SO^a^ (s)	HbR Min p	HbR FWER	HbR Nominal (p < 0.05)	HbO PAI of min p channel^b^	HbO CG→SO^a^ (s)	HbO Min p	HbO FWER	HbO Nominal (p < 0.05)	Seizure	Patient
1,343,731	442.9	0.010	0	2	970,526	124.0	0.031	0	1	1	P01
650,794	223.6	0.057	0	0	1,180,013	75.8	0.042	0	1	2	P01
362,407	395.0	0.162	0	0	1,204,150	326.4	0.061	0	0	1	P02
230,372	438.6	0.122	0	0	764,177	202.3	0.064	0	0	2	P02
212,493	474.0	0.188	0	0	174,595	405.0	0.217	0	0	1	P03
1,005,894	279.3	0.058	0	0	320,552	349.8	0.162	0	0	1	P06
1,325,638	139.1	0.050	0	1	2,369,525	87.6	0.024	0	2	2	P06
900,949	363.3	0.056	0	0	1,088,459	341.9	0.038	0	1	3	P06
1,918,563	203.6	0.034	0	1	2,324,077	192.8	0.035	0	4	4	P06
10,232,459	150.1	0.003	0	3	4,596,121	442.7	0.011	0	7	6	P06
1,896,432	20.3	0.025	0	1	5,507,341	394.3	0.008	0	4	1	P07
505,844	156.1	0.104	0	0	423,494	404.8	0.119	0	0	2	P07
544,698	196.0	0.114	0	0	490,250	632.1	0.111	0	0	3	P07
115,979	592.0	0.223	0	0	1,037,640	244.4	0.046	0	1	4	P07
8,874,851	73.3	0.008	0	3	45,049,560	72.0	0.0005	1	2	6	P07
3,455,177	518.0	0.008	0	4	751,476	216.4	0.067	0	0	7	P07

Abbreviations: FWER, family-wise error rate correction; Min p, smallest uncorrected empirical p-value observed across channels for that seizure and chromophore

a CG→SO – center of gravity to seizure onset was calculated for the channel with the minimum p value for that seizure and chromophore

b PAI (pre-ictal activity index) for the channel with the minimum uncorrected empirical *p*-value for that seizure and chromophore

#### 3.3.1 Patient 01

In P01, nominally significant channel-level findings were observed in both chromophores. HbO demonstrated one nominally significant channel in each of the two analyzable seizures. HbR demonstrated two nominally significant channels in seizure 1 and one nominally significant channel in seizure 2. No individual channel survived FDR or FWER correction.

#### 3.3.2 Patients 02 and 03

P02 and P03 demonstrated little evidence of seizure-associated ultra-slow activity. No channels survived multiple-comparison correction. Although two channel-level effects in P02 approached nominal significance (uncorrected p ≈ 0.06), no nominally significant findings were observed in either patient. These patients therefore served as negative examples within the cohort.

#### 3.3.3 Patient 06

Among the five analyzable HbO seizures, four (80%; seizures 2, 3, 4 and 6) demonstrated nominally significant channel-level findings. The number of nominally significant channels ranged from 1 to 7 across these seizures, with seizure 6 showing seven channels below the uncorrected threshold (minimum empirical p = 0.011). HbR demonstrated a similar pattern. Nominally significant channels were observed in seizures 2, 4 and 6, with seizure 6 again producing the largest number of significant channels.

When HbO and HbR were considered jointly, ten nominally significant channels were observed in seizure 6. Despite the repeated occurrence of low p-values across multiple seizures, no individual channel survived FDR or FWER correction.

#### 3.3.4 Patient 07

P07 demonstrated the strongest individual channel-level finding in the cohort. In HbO, seizure 6 produced a highly significant focal effect centered on channel 10. The minimum empirical p-value was approximately 0.0005 and survived both FDR and FWER correction.

Additional nominally significant channels were observed in seizures 1, 4 and 6. HbR findings were generally weaker but demonstrated a similar overall distribution across seizures.

Taken together, these findings indicate that seizure-associated ultra-slow activity was detectable at the individual-channel level in a subset of patients, with the strongest focal evidence observed in P07 and the strongest repeated evidence observed in P06.

### 3.4 Temporal characteristics of pre-ictal activity

To characterize the timescale of the detected hemodynamic activity, CG-to-seizure-onset durations were examined for the channel demonstrating the lowest empirical p-value within each seizure and chromophore (Table 2, and Fig. 2C–D). For HbO, durations ranged from 72.0 to 632.1 s (median = 280.4 s), whereas HbR durations ranged from 20.3 to 592.0 s (median = 241.5 s). Overall, these intervals were typically several minutes before SEEG-defined seizure onset, indicating that the detected activity frequently contained a substantial pre-ictal component, consistent with the hypothesis that seizure-associated hemodynamic dynamics may evolve well before electrographic seizure onset.

### 3.5 Seizure-level Fisher analysis

**Fig. 3:**
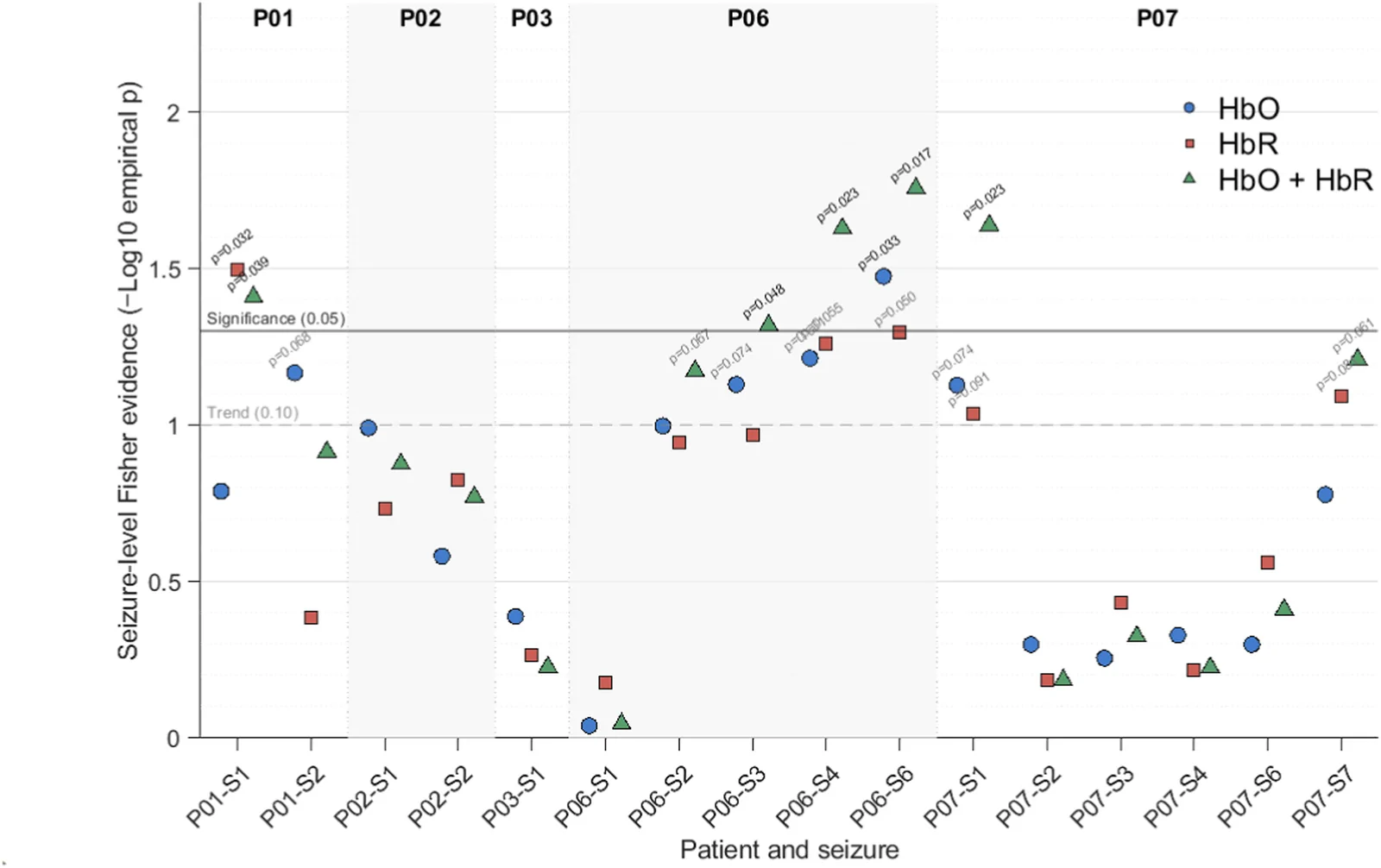
**Seizure-level Fisher evidence across chromophores.** Seizure-level Fisher p-values are shown for HbO, HbR, and combined HbO + HbR analyses for all analyzable seizures. Values are displayed as −log_10_(p), such that larger values indicate stronger evidence for an excess of low channel-level p-values relative to the permutation-derived null distribution. The solid horizontal line denotes the conventional significance threshold (p = 0.05), and the dashed horizontal line denotes trend-level significance (p = 0.10). Each point represents a single seizure analysis. Significant and trend-level findings were observed in several seizures from P01, P06 and P07, whereas Fisher statistics for P02 and P03 generally remained within the range expected under the null model.

Because seizure-related effects may be distributed across multiple channels without surviving correction at any individual channel, channel-level p-values were aggregated within seizures using Fisher's method (Fig. 3).

#### 3.5.1 Patient 01

P01 demonstrated significant seizure-level Fisher evidence in HbR seizure 1 (p = 0.032). HbO seizure 1 demonstrated trend-level significance (p = 0.068). No significant combined HbO + HbR Fisher findings were observed.

#### 3.5.2 Patients 02 and 03

No significant seizure-level Fisher findings were observed in P02 or P03. Fisher statistics remained within the range expected under the permutation-derived null distributions for HbO, HbR and combined analyses.

#### 3.5.3 Patient 06

P06 demonstrated the strongest and most consistent seizure-level Fisher evidence in the cohort. In the combined HbO + HbR analysis, seizures 3, 4 and 6 were significant (p = 0.048, p = 0.024 and p = 0.017, respectively).

For HbO alone, seizure 6 reached significance (p = 0.033), while seizures 3 and 4 demonstrated trend-level findings (p = 0.074 and p = 0.061, respectively). For HbR, seizure 6 reached the threshold for significance (p = 0.050), and seizure 4 demonstrated trend-level significance (p = 0.055).

These findings indicate that multiple P06 seizures contained a greater burden of significant channel-level p-values than expected under the permutation-derived null distribution, even when individual channels did not survive strict correction for multiple comparisons.

#### 3.5.4 Patient 07

In HbO, no seizures reached significance in the seizure-level Fisher analysis. Seizure 1 demonstrated trend-level significance (p = 0.074), whereas the remaining seizures were non-significant. In HbR, no seizures reached significance. Seizures 1 and 7 demonstrated trend-level significance (p = 0.091 and p = 0.081, respectively), while all other seizures were non-significant.

In the combined HbO + HbR analysis, seizure 1 demonstrated significant Fisher evidence (p = 0.023), and seizure 7 demonstrated trend-level significance (p = 0.061). No other combined analyses reached significance.

Overall, seizure-level Fisher analysis provided limited but detectable evidence of seizure-associated ultra-slow activity in P07. Notably, the strongest evidence emerged in the combined HbO + HbR analysis rather than in either chromophore alone.

### 3.6 Spatial distribution of nominal effects

**Fig. 4:**
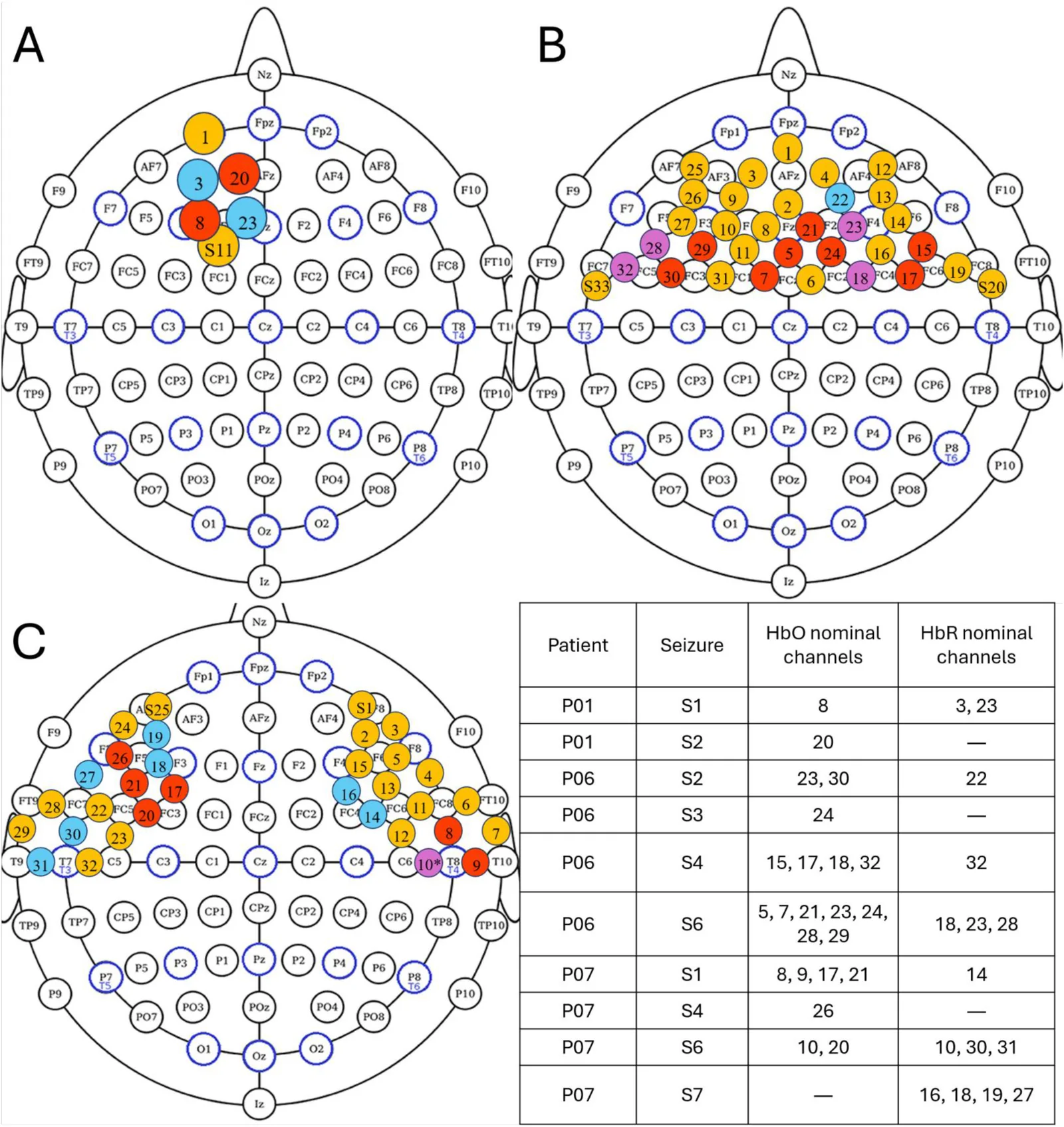
**Spatial distribution of nominally significant channel-level findings and seizure-specific channel involvement.** Panels A–C show the fNIRS channel layouts for patients P01, P06 and P07, respectively. Channels are highlighted if they demonstrated nominal significance (uncorrected empirical p < 0.05) in at least one analyzable seizure. Channels demonstrating nominal HbO, HbR or combined HbO + HbR findings are shown in red, blue and purple, respectively. Short-separation channels are identified by the prefix "S". Channel 10 in patient P07 (panel C) was the only channel-level finding to survive family-wise error correction and is marked with an asterisk. Panel D summarizes the nominally significant long-separation channels identified in each seizure for HbO and HbR.

The spatial distribution of nominally significant channels was examined to determine whether seizure-associated findings were randomly distributed across the recording montage or demonstrated spatial organization. The location of these channels was also compared with the clinically and electrophysiologically hypothesized seizure onset zones and seizure networks (Fig. 4).

#### 3.6.1 Patient 01

P01 was monitored using a lower-density montage comprising five long-separation channels. Nominally significant findings were observed in channels 8 and 20 for HbO and channels 3 and 23 for HbR. These findings were located within the frontal region sampled by the montage and were not inconsistent with the left frontal localization suggested by clinical and electrophysiological data. However, the limited spatial coverage precluded strong conclusions regarding spatial organization.

#### 3.6.2 Patient 06

Nominally significant findings in P06 demonstrated a non-uniform spatial distribution. Several neighboring channels were implicated within the same seizures, including channels 15, 17 and 18 in seizure 4 and channels 21, 23, 24, 28 and 29 in seizure 6. Channels 23 and 24 were involved across multiple seizures. The observed distribution was concentrated within anterior frontal regions and broadly corresponded to the mediofrontal and orbitofrontal seizure network identified by SEEG.

#### 3.6.3 Patient 07

P07 demonstrated a similarly organized spatial pattern. Neighboring channels were implicated within individual seizures, including channels 16, 18, 19 and 27 in seizure 7 and channels 10, 20, 30 and 31 in seizure 6. The strongest channel-level finding in the cohort occurred in channel 10 during seizure 6 and survived family-wise correction. The overall distribution was concentrated within right temporal and frontotemporal regions and broadly corresponded to the suspected right temporal seizure localization.

Taken together, nominally significant findings were not distributed uniformly across the recording montages. Instead, they tended to occur within restricted anatomical regions and frequently involved neighboring channels within the same seizure. Although these observations do not establish a cortical origin of the detected activity, they are consistent with spatially organized physiological processes and, in P06 and P07, broadly corresponded to the seizure localization suggested by independent clinical and electrophysiological assessments.

#### 3.6.4 Comparison with short-separation channels

To evaluate whether the observed findings could be explained by extracerebral physiological fluctuations, the same analytical framework was applied to short-separation channels.

**Fig. 5:**
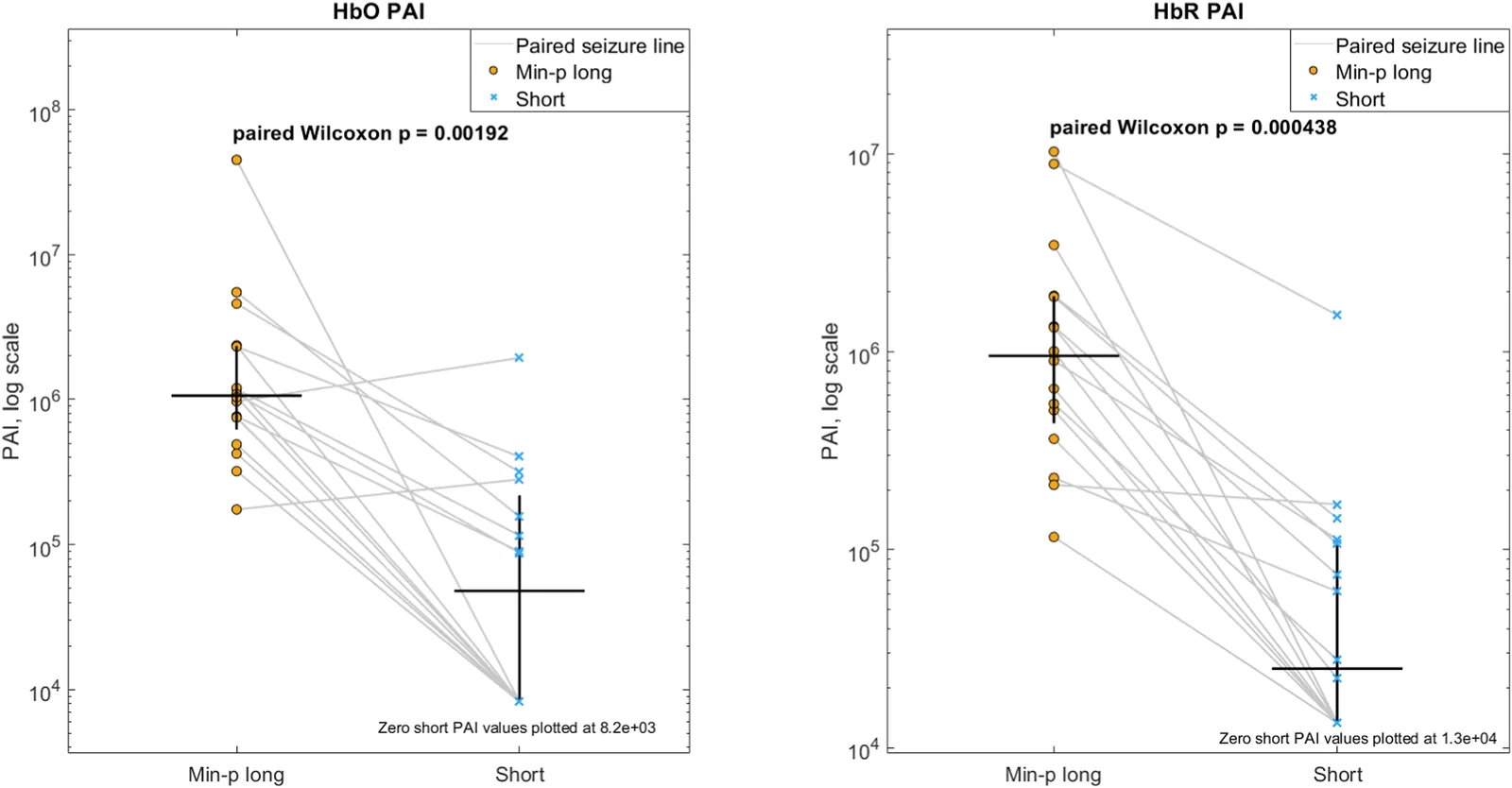
**Comparison of pre-ictal activity (PAI) between long- and short-separation channels.** For each analyzable seizure and chromophore, the long-separation channel with the minimum uncorrected empirical *p*-value (Min-p long) was compared with the nearest short-separation channel (Short). Orange circles indicate the selected long-separation channels, blue crosses indicate the corresponding short-separation channels, and grey lines connect paired measurements from the same seizure. Black horizontal and vertical bars represent the median and interquartile range, respectively. PAI values are displayed on a logarithmic scale. Paired Wilcoxon signed-rank tests demonstrated larger PAI values in long-separation than short-separation channels for both HbO and HbR. Zero PAI values were displayed at the smallest non-zero PAI value; this adjustment was applied for display purposes only and was not used in the statistical analyses.

Comparison of the long-separation channel demonstrating the minimum empirical p-value with the corresponding nearest short-separation channel revealed substantially larger PAI values in the long-separation channels for both HbO and HbR (Fig. 5). Across seizure–chromophore pairs, long-separation PAI values were significantly greater than those observed in the corresponding short-separation channels (paired Wilcoxon signed-rank tests: HbO, p = 0.0019; HbR, p = 0.0004). Although extracerebral contributions cannot be excluded, these findings, together with the spatial clustering of nominally significant channels, suggest that the observed long-separation effects cannot be fully explained by superficial physiological fluctuations alone.

### 3.7 Convergence of HbO and HbR findings

Although channel-level effects were generally stronger in HbO, similar patterns were observed in HbR. In P06, nominal HbR findings occurred in several of the same seizures that demonstrated HbO findings, and seizure-level Fisher significance was strengthened when both chromophores were analyzed jointly.

The strongest seizure-level Fisher results in the study were observed in the combined HbO + HbR analyses of P06, where three seizures reached statistical significance. This convergence across chromophores supports the interpretation that the observed phenomenon reflects coordinated hemodynamic activity rather than isolated fluctuations in a single signal.

### 3.8 Summary of findings

Within the defined 0.002–0.004 Hz analysis band, the analyses revealed convergent evidence for seizure-associated ultra-slow hemodynamic activity within the 0.002–0.004 Hz frequency range.

Overall, evidence for seizure-associated ultra-slow hemodynamic activity, defined as significant seizure-level Fisher analysis or nominally significant channel-level findings, was identified in three of the five analyzed patients and in 10 of the 16 analyzable seizures. Evidence was strongest in P06, where repeated seizure-level Fisher findings, spatial clustering of nominal effects and concordant HbO and HbR activity were observed across multiple seizures. P07 demonstrated the strongest focal finding in the cohort, including the only channel-level result to survive family-wise correction. P01 demonstrated weaker but significant seizure-level Fisher evidence, whereas P02 and P03 showed little evidence of seizure-associated activity.

The combination of channel-level permutation testing, seizure-level Fisher analysis, spatial concentration of nominal findings, convergence across chromophores and limited correspondence with short-separation channels supports the presence of seizure-associated ultra-slow hemodynamic changes in a subset of patients.

## 4 Discussion

The principal finding of this study is that very low-frequency hemodynamic oscillations occurred preferentially around SEEG-defined seizure onsets compared with pseudo-seizure events sampled from the same recordings. Although the findings were heterogeneous across patients, the convergence of permutation testing, seizure-level analyses, and spatial organization provides preliminary evidence that ultra-slow hemodynamic dynamics may be associated with the pre-ictal state.

The present analyses were restricted to a single exploratorily defined analysis band (0.002–0.004 Hz). This exploratory step was intended to define the scope of formal statistical testing rather than to establish the biological importance of this frequency range. Although this range was selected through an exploratory procedure and therefore requires independent validation, the convergence of findings across seizures and patients suggests that extremely slow hemodynamic processes may represent a particularly informative component of peri-ictal physiology. Previous electrophysiological studies have implicated infra-slow activity and slowly evolving changes in cortical excitability preceding seizure onset (Constantino and Rodin, 2012; Hashimoto et al., 2021; Lee et al., 2022). However, the frequency range identified in the present study lies at the extreme low end of those typically examined and corresponds to physiological fluctuations evolving over several minutes. While the present study does not establish a direct relationship between these hemodynamic oscillations and infra-slow electrophysiological processes, the findings raise the possibility that seizure-related dynamics may extend to even slower timescales than those commonly investigated in electrophysiological studies.

The temporal characteristics of the detected activity are also noteworthy. Among seizures demonstrating the strongest effects, CG-to-seizure-onset intervals frequently ranged from approximately one to 7 min. Because CG is a conservative descriptor of the temporal distribution of wavelet power, these intervals should not be interpreted as direct estimates of the onset of the underlying physiological process and likely represent lower-bound estimates of the duration of the pre-ictal component. Nevertheless, the finding that CG frequently preceded SEEG-defined seizure onset by several minutes indicates that the detected activity was often not confined to the immediate peri-onset period and contained a substantial pre-ictal component. This observation is broadly consistent with previous reports of slowly evolving physiological changes preceding seizure onset across multiple modalities (Baumgartner et al., 1998; Gadhoumi et al., 2016; Mormann et al., 2007; Vinette et al., 2016).

Ultra-low-frequency CWT analyses are also susceptible to edge effects (Torrence and Compo, 1998), which increase uncertainty in wavelet estimates near the temporal boundaries of the analyzed recordings (see *Supplementary* sections *I &II*). Although the exclusion of seizures occurring within 30 min of recording boundaries substantially reduces edge-related distortion within the analyzed peri-ictal windows, some residual uncertainty related to edge effects inherent to CWT cannot be completely excluded.

A major challenge in interpreting ultra-slow fNIRS activity is that similar fluctuations occur spontaneously throughout prolonged recordings and may arise from resting-state vascular oscillations, systemic physiological processes, or spontaneous neurovascular activity unrelated to seizures (Obrig, 2014; Scholkmann et al., 2014; Tong and Frederick, 2014). Consequently, the observation of peri-ictal fluctuations alone does not establish a seizure-related process. Accordingly, the permutation framework was designed to estimate how frequently comparable fluctuations would be expected elsewhere within the same recordings (Maris and Oostenveld, 2007; Nichols and Holmes, 2002). Several independent observations argue against a purely random or superficial explanation. First, multiple seizures exceeded permutation-derived null distributions generated from pseudo-seizure events. Second, significant and trend-level findings recurred across multiple seizures within individual patients, suggesting stable patient-specific phenomena rather than isolated events. Third, nominally significant channels frequently formed localized spatial clusters that broadly corresponded to independently defined seizure onset hypotheses, while the strongest long-separation findings were generally not mirrored by neighboring short-separation channels. Finally, although statistically significant findings were generally more prominent in HbO than HbR, this pattern is consistent with previous fNIRS epilepsy studies. At the same time, HbO is recognized to be more susceptible to extracerebral and systemic physiological influences (Tachtsidis and Scholkmann, 2016). The observation that several seizures demonstrated convergent findings across both chromophores, particularly in the combined Fisher analyses, provides additional support that the detected effects are not solely driven by isolated HbO fluctuations, while also reinforcing the need for cautious interpretation. Although none of these observations alone excludes systemic physiological contributions, together they provide convergent evidence that at least part of the detected activity reflects structured physiological processes temporally associated with seizure onset.

The physiological origin of these oscillations nevertheless remains uncertain, and temporal association with seizure onset should not be interpreted as evidence of a neuronal process. They may reflect gradual neurovascular changes accompanying the transition into a seizure state (Nguyen et al., 2012; Peng et al., 2016; Zhao et al., 2011), including evolving alterations in neuronal activity, vascular regulation, autonomic activity, metabolism, or neurovascular coupling (Pinti et al., 2020; Scarapicchia et al., 2017), or they may represent broader physiological states that modulate seizure susceptibility without directly participating in seizure generation. These possibilities are not mutually exclusive, and the present study cannot distinguish between them. It is noteworthy, however, that the studied frequency range lies well below the dominant frequencies of cardiac pulsation (∼1 Hz), respiration (∼0.2-0.4 Hz), and Mayer-wave oscillations (∼0.1 Hz). While very slow systemic and vascular processes remain plausible contributors, the findings are unlikely to be explained solely by simple contamination from these higher-frequency physiological sources.

An important consideration is the substantial difference in recording configurations across patients. The strongest findings were observed in P06 and P07, who were monitored using the NIRScout system with 30–31 long-separation channels and longer recording durations. In contrast, P01–P03 were recorded using the earlier Hitachi system with only five long-separation channels and generally shorter monitoring periods. In addition to differences in channel density and recording duration, the two systems also differed in acquisition characteristics, including emitted wavelengths, sampling frequency, and optode configuration, all of which may have influenced measurement sensitivity. Because acquisition system was completely confounded with participant identity, the present dataset does not allow hardware-related effects to be distinguished from true biological variability. Consequently, the absence of clear findings in P02 and P03 should not necessarily be interpreted as evidence that ultra-slow hemodynamic activity is absent in these patients, but may instead reflect reduced sensitivity arising from methodological differences. Future studies employing larger cohorts, standardized acquisition platforms, more standardized spatial coverage, and more extensive physiological monitoring will be important to clarify these effects and to further characterize the relationship between ultra-slow hemodynamic dynamics and the neuronal processes underlying seizure initiation.

The use of SEEG as the temporal reference point represents an important strength of the present study. Previous investigations of peri-ictal hemodynamics have frequently relied on scalp EEG or clinical seizure onset. Because intracranial recordings may detect seizure onset earlier than scalp recordings (Barborica et al., 2021), SEEG provides a more precise estimate of electrophysiological seizure initiation and reduces the likelihood that early ictal activity is misclassified as pre-ictal. The observation that pre-ictal ultra-slow activity could be detected using scalp-based fNIRS despite intracranially defined seizure onset suggests that these physiological processes may extend beyond the immediate seizure onset zone and may be accessible to non-invasive optical monitoring. The present findings should not be interpreted as demonstrating a clinically useful seizure prediction method. Rather, they suggest that ultra-slow hemodynamic activity contains information associated with the evolving pre-ictal state. Future studies integrating these physiological signals with electrophysiological and computational approaches may help determine whether they can improve seizure prediction, facilitate characterization of seizure-generating networks, or provide complementary physiological markers of the transition into the ictal state.

Several limitations should be acknowledged. The study included only five patients and sixteen analyzable seizures. Two different fNIRS systems were used, resulting in substantial differences in channel density, spatial coverage, recording characteristics, and acquisition parameters across patients. As a result, hardware-related effects could not be fully separated from biological variability, limiting interpretation of between-patient differences and the generalizability of the findings. Recording durations also varied considerably between patients, making it difficult to disentangle the relative contributions of spatial coverage and monitoring duration to the observed findings. The frequency range selected for formal testing was identified through exploratory inspection of seizure-averaged scalograms and therefore should be considered hypothesis-generating until validated in an independent cohort. Although the strongest long-separation findings were generally not mirrored by corresponding short-separation channels, the available short-separation data were insufficient to definitively determine the relative contributions of cortical, extracerebral and systemic physiological signals. Finally, the study focused exclusively on hemodynamic measurements and did not directly examine relationships between the observed oscillations and concurrent infra-slow electrophysiological activity.

More broadly, establishing the existence and characteristics of seizure-associated ultra-slow hemodynamic oscillations is relevant to the continued development of optical monitoring approaches for epilepsy. The limited spatial specificity and depth sensitivity of conventional scalp-based measurements have motivated efforts to develop optical technologies capable of more directly interrogating seizure-related hemodynamic processes. Computational modelling, phantom studies (Rein et al., 2024) and preliminary in-vivo investigations (Heymann et al., 2025) have suggested that advanced scalp-based optical systems as well as emerging intracranial optical monitoring approaches may provide improved sensitivity to deeper epileptic networks and more precise characterization of neurovascular dynamics. These findings therefore provide a physiological rationale for continued development of optical approaches capable of characterizing seizure-related vascular dynamics with greater sensitivity (Rein et al., 2025) and spatial specificity.

In conclusion, this study provides evidence that very low-frequency hemodynamic oscillations occur preferentially around SEEG-defined seizure onsets in a subset of patients with focal epilepsy. By comparing observed findings with permutation-derived pseudo-seizure events, the analysis suggests that these fluctuations cannot be fully explained by chance occurrences within prolonged recordings. Although the physiological origin and clinical significance of the observed oscillations remain uncertain and the identified frequency band should be considered exploratory pending independent validation, the temporal organization, spatial clustering, and frequent pre-ictal component of these oscillations support further investigation of ultra-slow hemodynamic dynamics as a potentially informative aspect of seizure-related physiology.

## CRediT authorship contribution statement

**Netaniel Rein:** Writing – review & editing, Writing – original draft, Visualization, Validation, Software, Project administration, Methodology, Investigation, Formal analysis, Data curation, Conceptualization. **Revital Shechter:** Writing – review & editing, Methodology, Data curation, Conceptualization. **Yael Avni:** Writing – review & editing, Resources, Data curation. **Evgeny Tsizin:** Writing – review & editing, Investigation, Data curation. **Oshrit Arviv:** Writing – review & editing, Investigation, Data curation. **Dana Ekstein:** Writing – review & editing, Methodology, Investigation, Data curation. **Tal Benoliel:** Writing – review & editing, Data curation. **Diya Doufish:** Writing – review & editing, Data curation. **Tal Gilboa:** Writing – review & editing, Data curation. **Naomi Froimovich:** Writing – review & editing, Data curation. **Yuliya Katz:** Writing – review & editing, Data curation. **Paz Har-Shai Yahav:** Writing – review & editing, Investigation. **Guy Rosenthal:** Writing – review & editing, Data curation. **Sami Heymann:** Writing – review & editing, Data curation. **Zvi Israel:** Writing – review & editing, Methodology, Data curation. **Michal Balberg:** Writing – review & editing, Visualization, Validation, Supervision, Software, Resources, Project administration, Methodology, Investigation, Funding acquisition, Formal analysis, Data curation, Conceptualization. **Mordekhay Medvedovsky:** Writing – review & editing, Writing – original draft, Visualization, Validation, Supervision, Software, Resources, Project administration, Methodology, Investigation, Funding acquisition, Formal analysis, Data curation, Conceptualization.

## Declaration of generative AI in the writing process

During the preparation of this manuscript, the authors used generative AI tools to improve the readability and language of the text. After using these tools, the authors reviewed and edited the content as required and take full responsibility for the content of the publication.

## Funding

This work was funded by the Israeli Innovation Authority under grants numbers 77084 and 77092.

## Declaration of competing interest

The authors declare the following financial interests/personal relationships which may be considered as potential competing interests:Netaniel Rein, Mordekhay Medvedovsky, Michal Balberg, Revital Shechter, and Yael Avni are inventors on pending patent application PCT/IL2024/051091, entitled “Systems for Applying Functional Near Infra-red Spectroscopy (fNIRS) to a Head of a Subject.” Michal Balberg, Mordekhay Medvedovsky, and Evgeny Tsizin are inventors on pending patent application EP23823400.9A, entitled “Multimodal Functional Brain Sensor.” Both applications were filed by Hadasit Medical Research Services and Development Ltd. and A.Y.Y.T. – Technological Applications and Data Update Ltd. The other authors declare no competing financial interests or personal relationships that could have appeared to influence the work reported in this paper.

## Data Availability

The data that support the findings of this study are available from the corresponding author upon reasonable request.
