# Supplementary material for: Functional near-infrared spectroscopy pre-ictal oscillations recorded simultaneously with stereo-EEG
Source: Neuroimage Rep. 2026 Sep 10;6(4):100401. doi: 10.1016/j.ynirp.2026.100401 (PMC13586569; doi:10.1016/j.ynirp.2026.100401)
Supplement: Supplementary file 1 [file mmc1.docx]

**Supplementary**

**Contents –**

I) Edge artifact
II) Simulation of low-frequency temporal smearing in continuous wavelet transform analysis
III) Montages of patients 2 and 3
I) **Edge artifact**

In CWT, edge artifacts occur near the beginning and end of a signal. These regions are less reliable because the wavelet, which is convolved with the signal, extends beyond the signal’s boundaries, resulting in distortions due to zero-padding or extrapolation. Such edge regions may exhibit spurious high or low power that does not reflect true signal dynamics, potentially leading to false positives if not interpreted cautiously[1].

When CWT is implemented in MATLAB, a cone of influence (COI) is plotted to visually indicate regions where edge effects may distort the results (Fig. Supp. 1). MATLAB estimates the COI based on the temporal support of the wavelet at each frequency. Larger wavelets, used for lower frequencies, extend farther in time, increasing the size of the unreliable boundary region.

Because the present study focused on ultra-low-frequency oscillations (0.002–0.004 Hz), the corresponding wavelets have a long temporal support, resulting in a particularly wide cone of influence. As shown in Figure Supp. 1, the COI extends to more than 15 minutes from each signal boundary at the lowest frequencies analyzed. Consequently, apparent power changes occurring within these regions may reflect boundary artifacts rather than genuine physiological activity. To minimize this risk, all analyses were designed to avoid interpreting CWT features occurring within the COI, and seizures or pseudo-onsets for which the required peri-ictal window extended into these unreliable boundary regions were excluded from further analysis.

**Fig Supp 1 -** Scalogram of the Continuous Wavelet Transform (CWT) from one patient, with the cone of influence (COI) indicated by a white dashed line. The shaded area represents regions of the signal affected by edge artifacts, where data is considered unreliable.

II) **Simulation of low-frequency temporal smearing in continuous wavelet transform analysis**

CWT is often used in fNIRS at higher frequencies of ~0.02-0.1Hz, which are higher than those relevant to this study. Although edge artifacts can be addressed by excluding the cone of influence, a separate challenge arises at very low frequencies. Even well away from signal boundaries, increases in CWT power appear temporally blurred, making the precise onset of activity difficult to determine. In order to determine whether the slow increase in power was solely caused by slow changes in cerebral hemodynamics, or whether it was an artifact of CWT we ran a simulation using the code below (Fig supp 2).

 **Fig Supp 2** – MATLAB code used to generate the simulated signal for evaluation of low-frequency CWT timing.

Simulated activity comprising waves across five orders of magnitude with a discrete onset time was generated. CWT analysis showed increasing blurriness at lower orders of magnitude (Fig Supp 3), indicating that the blurred edges of activity changes observed at lower frequencies were partly due to the CWT process itself and not caused solely by hemodynamic changes. These blurred edges complicated the determination of the precise onset of hemodynamic changes at lower frequencies.

To determine whether a robust timing metric could be identified despite this temporal smearing, we evaluated the behavior of the center of gravity (CG) of peri-ictal wavelet power. CG lagged behind the true onset of activity in the simulation. While this approach could lead to underestimation of pre-ictal detection times, the focus of our study on pre-ictal activity detection prompted a conservative stance, prioritizing the avoidance of false positives over false negatives.

**Fig Supp 3.** Continuous wavelet transform (CWT) of the artificial signal **a** defined in **Fig. S2**. The y-axis shows frequency (Hz) on a logarithmic scale, the x-axis shows time (samples), and the color scale represents wavelet power. For each frequency bin, the detected blob characteristics are overlaid: blob onset and end are indicated by cyan vertical markers, the interquartile range (IQR) start and end by green vertical markers, and the center of gravity (CG) by red points. Two key observations emerge. First, for frequencies with a well-defined increase in power, the CG consistently occurs after the true onset of the simulated signal, reflecting the progressive accumulation of power following onset. Second, at very low frequencies (e.g., approximately $1.5\times{10}^{-4}$Hz), the temporal smearing inherent to the CWT causes the leading edge of the wavelet representation to extend before the true signal onset (green arrow), producing an apparent pre-onset increase in power despite the absence of signal before onset. Importantly, although this low-frequency smearing substantially biases estimates based on the earliest detectable activity or blob onset, it has only a limited effect on the CG, which remains well within the true period of signal activity. These findings demonstrate that, although the earliest detectable wavelet activity is substantially advanced by low-frequency temporal smearing, the CG remains centered within the true period of signal activity. Consequently, the CG provides a conservative timing metric that is substantially less susceptible to CWT-induced temporal bias than onset-based measures.

III) **Montages of patients 2 and 3**

**Figure Supp. 4**. fNIRS montages for Patients P02 (A) and P03 (B). Yellow circles indicate long-separation channels included in the statistical analyses, and S11 denotes the short-separation channel. These montages are provided to illustrate channel placement relative to the scalp and complement the patient-specific analyses presented in the main manuscript.
